# Quantitative inner membrane proteome datasets of the wild-type and the Δ*min* mutant of *Escherichia coli*

**DOI:** 10.1016/j.dib.2016.05.043

**Published:** 2016-05-27

**Authors:** Suh-Yuen Liang, Shu-Yu Lin, I-Chen Chiang, Yu-Ling Shih

**Affiliations:** a Institute of Biological Chemistry, Academia Sinica, Taipei 115, Taiwan; b Institute of Biochemical Sciences, National Taiwan University, Taipei 106, Taiwan; c Department of Microbiology, College of Medicine, National Taiwan University, Taipei 100, Taiwan

**Keywords:** *Escherichia coli*, The Min system, Inner membrane protein, Mass spectrometry, iTRAQ

## Abstract

This article presents data that were obtained through measuring the impact of the Min oscillation on membrane proteins in *Escherichia coli* by quantitative protemoics analysis. We isolated inner membranes from the wild-type and mutant strains to generate proteomics datasets based on NanoLC-nanoESI-MS/MS mass spectrometry using the isobaric tags for relative and absolute quantitation (iTRAQ) method. The datasets included the raw spectral files from four sample replicates and the processed files using Proteome Discoverer that contained a total of 40,072 MS/MS spectra with confident peptide identifier (FDR<0.01) and the peak intensity of the reporter ions. The data was further filtered, which resulted in an inner membrane proteome of unique proteins with quantitation. Proteins of interest, that show significant difference in protein abundance of the mutant membrane, were isolated through statistical filtering. The data is related to “**Quantitative proteomics analysis reveals the Min system of*****Escherichia coli*****modulates reversible protein association with the inner membrane**” (Lee et al., 2016 [1]).

**Specifications Table**

**Table:** 

Subject area	Biology
More specific subject area	Bacteria, cell division, protein-membrane interaction
Type of data	Table, Excel file
How data was acquired	NanoLC-nanoESI-MS/MS
Data format	Raw
Experimental factors	The Min proteins
Experimental features	Inner membrane proteins of the wild-type and the Δ*min* mutants of *Escherichia coli* were subjected to iTRAQ labeling and mass spectrometry analysis. The data were processed and statistically filtered to obtain an inner membrane proteome and to identify proteins of interest for functional analysis.
Data source location	Institute of Biological Chemistry, Academia Sinica, Taipei, Taiwan
Data accessibility	Data are available within the article and are deposited to the ProteomeXchange Consortium via the Proteomics Identifications (PRIDE) partner repository with the dataset identifier PRIDE: PXD002548.

## Value of the data

• Data provide an inner membrane proteome of *E. coli* that contains information of protein topology and identifies new membrane-associating proteins.
• The iTRAQ datasets offer the signature peptides with spectral evidence for the inner membrane proteins of the Δ*min* mutant, which could be useful for the experimental design of the targeted quantitation of proteins by mass spectrometry.
• Data show enrichment in peripheral membrane proteins and metabolic enzymes that could represent a correlation between protein topology and function.
• The network created based on the proteins of interest could guide assessment of molecular and genetic interactions to uncover additional function of the Min system.

## 1 Data

The mass spectrometry data files were generated to address whether the membrane-associating oscillator of the *E. coli* Min proteins would have a general role in cell physiology by influencing topology of membrane proteins [1]. The presented data include the dataset files (noted in Supplementary Table 1), the combined peptide quantification file (Supplementary Table 2), a network built based on the filtered proteins of interest (Network 1), and a network of MinD and MinE (Network 2).

## 2 Experimental design, materials and methods

### 2.1 Isolation of inner membrane

We studied the wild-type *E. coli* strain MC1000 [*F-araD139 Δ(araABO1C-leu) galUK Δ(lacZYAI)x74 rpsL thi leu lac*] [2] and the Δ*min* mutant strains YLS1 [MC1000 Δ*min* Cm^R^] [3] and RC1 [MC1000 *min:aph*] [4]. We isolated the inner membrane (IM) by disruption of spheroplasts followed by sucrose-gradient ultracentrifugation [5]. The IM fractions were confirmed by the NADH oxidase activity.

### 2.2 Labeling with isobaric tags for relative and absolute quantitation

We resolved the IM proteins on SDS-PAGE (4% stacking and 10% separation gels) for protein extraction. The proteins were reduced, cysteine blocked, and digested, before labeling with the iTRAQ® reagent (Roche, Basel, Switzerland) containing different isobaric tags (114, 115, 116, or 117) according to the manufacturer׳s instruction. Four sample replicates were prepared from protein digests derived from independent cultures; each was labeled with a different isobaric tag. The protein samples from different strains with different isobaric tags were mixed in equal amounts for the LC–MS/MS analysis (Supplementary Table 1).

### 2.3 Mass spectrometry

A nanoAcquity system (Waters, MA, USA) was connected to the Orbitrap Elite hybrid mass spectrometer (Thermo Electron, Bremen, Germany) equipped with a PicoView nanospray interface (New Objective, MA, USA) for the NanoLC-nanoESI-MS/MS analysis. A 75 μm inner diameter, 25 cm length C18 BEH column (Waters) packed with 1.7 μm particles with a pore width of 130 Å was used for elution. Operation of the experiments was described in [1].

### 2.4 Protein quantitation and statistical analysis

We analyzed the mass spectrometry raw data with the Proteome Discoverer™ Software (v 1.4.1.14; Thermo Fisher Scientific, Inc.) and used Mascot (v 1.4; Matrix Science, MA, USA) for protein identification. The parameters used for Mascot processing were: precursor mass tolerance window of 10 ppm, fragment tolerance window of 50 mmu, dynamic carbamidomethylation on cysteine, dynamic oxidation on methionine, dynamic N-terminal and cysteine iTRAQ labeling. The missed cleavage was set as 2. We used all peptides of the first rank in each spectrum with a Mascot significant threshold less than 0.05 for data analysis to limit the false discovery rate (FDR) based on the target-decoy database algorithm. The raw intensity values of each reporter ion for all confident peptides, i.e. FDR<0.01, were exported from Proteome Discoverer and combined for further data processing by normalization and aggregation. The resulting datasets correlated to 4 sample replicates (Supplementary Table 1) and the quantification data of all replicates were combined in a file (Supplementary Table 2). The data were further filtered to obtain an IM proteome of 808 unique proteins with quantitation [1]. In Lee et al. [1], we compared the level of protein expression between two subjects in the datasets (YLS1 vs. MC1000 with different isobaric labels) by the paired-sample t test to examine if the mean difference between experimental subjects is different than 0. A cut-off ratio at ≥0.5 and a *p* value at ≤0.05 were applied to obtain 40 proteins of interest (POIs) [1]. Forty POIs were imported into Cytoscape (Version 3.1.0, supported by NIGSM & NRNB, US) to retrieve data for network analysis [1]. The refined network is presented as Network 1. The data relating to MinD and MinE were extracted to create Network 2.

### 2.5 Reference library of protein subcellular topology

The subcellular topology data from databases of STEP (Subcellular Topologies of *E. coli* Polypeptides) [6], PSORT (a database of protein subcellular localizations for bacteria and archaea) [7], ASKA clone (–) (A complete Set of *E. coli* K-12 ORF Archive; National BioResource Project, Japan), and Dynamic Localizome [8] were combined into the protein library of *E. coli* strain K12 (organism ID 83333, retrieved from UniProtKB) that was manually curated to exclude duplicates, disrupted proteins, pseudogenes, and mobile elements [1]. This protein library was used as the reference to refine the datasets and to search for protein topology.

## Appendix A Supplementary material

Supplementary material: **Table 1.** Information of the dataset files. **Table 2.** The peptide quantification file combines the four search result files as listed in Supplementary Table 1. **Network 1.** Network built based on the filtered proteins of interest. Under the PSIMI25 visual style, the red (positive value) to blue (negative value) shade is applied to the nodes according to the ratio of protein abundance on the inner membrane of the mutant. The thickness of the edges is drawn according to the mentha scores. **Network 2.** Network of MinD and MinE. Under the PSIMI25 visual style, the red (positive value) to blue (negative value) shade is applied to the nodes according to the ratio of protein abundance on the inner membrane of the mutant. The thickness of the edges is drawn according to the mentha scores.

## Footnotes

Appendix A Supplementary data associated with this article can be found in the online version at doi:10.1016/j.dib.2016.05.043.
