# Supplementary material for: Quantitative inner membrane proteome datasets of the wild-type and the Δmin mutant of Escherichia coli
Source: Data Brief. 2016 May 27;8:304–7. doi: 10.1016/j.dib.2016.05.043 (PMC4906036; doi:10.1016/j.dib.2016.05.043)
Supplement: Supplementary file 2 — Supplementary material: Table 1. Information of the dataset files. Table 2. The peptide quantification file combines the four search result files as listed in Supplementary Table 1. Network 1. Network built based on the filtered proteins of interest. Under the PSIMI25 visual style, the red (positive value) to blue (negative value) shade is applied to the nodes according to the ratio of protein abundance on the inner membrane of the mutant. The thickness of the edges is drawn according to the mentha scores. Network 2. Network of MinD and MinE. Under the PSIMI25 visual style, the red (positive value) to blue (negative value) shade is applied to the nodes according to the ratio of protein abundance on the inner membrane of the mutant. The thickness of the edges is drawn according to the mentha scores. [file mmc2.zip › Table/Supplementary Table 1.docx]

**Supplementary Table 1.** Information of the dataset files.

| **PRIDE Project PXD002548** | | **Remarks** | | |
| --- | --- | --- | --- | --- |
| **Search Files^1^ (.msf)** | **Raw Files (.raw)** | **Sample Replicate** | ***E. coli* Strain** | **iTRAQ Tag** |
| 2014-0403_iTRAQ-1 | 2014-0403_iTRAQ-1 | 1 | MC1000 | 114 |
|  |  |  | YLS1 | 115 |
|  |  |  | RC1 | 116 |
| 2014-0403_iTRAQ-2 | 2014-0403_iTRAQ-2 | 2 | MC1000 | 115 |
|  |  |  | YLS1 | 116 |
|  |  |  | RC1 | 117 |
| 2014-0403_iTRAQ-3 | 2014-0403_iTRAQ-3 | 3 | MC1000 | 116 |
|  |  |  | YLS1 | 117 |
|  |  |  | RC1 | 114 |
| 2014-0403_iTRAQ-4 | 2014-0403_iTRAQ-4 | 4 | MC1000 | 117 |
|  |  |  | YLS1 | 114 |
|  |  |  | RC1 | 115 |

^1^ Processed by Proteome Discoverer
